# HPV, tumour metabolism and novel target identification in head and neck squamous cell carcinoma

**DOI:** 10.1038/s41416-018-0364-7

**Published:** 2019-01-17

**Authors:** Jason C. Fleming, Jeongmin Woo, Karwan Moutasim, Massimiliano Mellone, Steven J. Frampton, Abbie Mead, Waseem Ahmed, Oliver Wood, Hollie Robinson, Matthew Ward, Christopher H. Woelk, Christian H. Ottensmeier, Emma King, Dae Kim, Jeremy P. Blaydes, Gareth J. Thomas

**Affiliations:** 1 Cancer Sciences, Faculty of Medicine, University of Southampton, Southampton, UK; 2 St. George’s University Hospitals NHS Foundation Trust, Tooting, London, UK; 3 Clinical and Experimental Sciences, Faculty of Medicine, University of Southampton, Southampton, UK

**Keywords:** Head and neck cancer, Cancer metabolism, Cancer imaging, Oral cancer

## Abstract

**Background:**

Metabolic changes in tumour cells are used in clinical imaging and may provide potential therapeutic targets. Human papillomavirus (HPV) status is important in classifying head and neck cancers (HNSCC), identifying a distinct clinical phenotype; metabolic differences between these HNSCC subtypes remain poorly understood.

**Methods:**

We used RNA sequencing to classify the metabolic expression profiles of HPV^+ve^ and HPV^−ve^ HNSCC, performed a meta-analysis on FDG-PET imaging characteristics and correlated results with in vitro extracellular flux analysis of HPV^−ve^ and HPV^+ve^ HNSCC cell lines. The monocarboxylic acid transporter-1 (MCT1) was identified as a potential metabolic target and tested in functional assays.

**Results:**

Specific metabolic profiles were associated with HPV status, not limited to carbohydrate metabolism. There was dominance of all energy pathways in HPV-negative disease, with elevated expression of genes associated with glycolysis and oxidative phosphorylation. In vitro analysis confirmed comparative increased rates of oxidative phosphorylation and glycolysis in HPV-negative cell lines. PET SUV(max) scores however were unable to reliably differentiate between HPV-positive and HPV-negative tumours. MCT1 expression was significantly increased in HPV-negative tumours, and inhibition suppressed tumour cell invasion, colony formation and promoted radiosensitivity.

**Conclusion:**

HPV-positive and negative HNSCC have different metabolic profiles which may have potential therapeutic applications.

## Introduction

The identification of common links between oncogenes and important regulators of metabolism has fuelled a resurgent interest in cancer cell metabolism, despite the Warburg effect being first described nearly a century ago.^1^ This observation, that cancer cells preferentially metabolise glucose even in the presence of abundant oxygen, underpins the common clinical use of positron emission tomography (PET) in clinical oncology; this imaging modality uses a glucose analogue, fluorine-18- fluorodeoxyglucose, to identify tissue with rapid glucose uptake. Aerobic glycolysis is an inefficient pathway for generating ATP, but is thought to confer a growth advantage for tumour cells, providing a biosynthetic advantage by generating carbon precursors required for the synthesis of macromolecules essential for cell division.^2,3^ While aerobic glycolysis has become a recognised ‘hallmark of malignancy’, its causal relationship to tumourigenesis remains unclear.

Head and neck squamous cell carcinoma (HNSCC) is currently classified into human papillomavirus-negative (HPV^−ve^) and -positive (HPV^+ve^) disease. HPV^+ve^ tumours are most commonly found in the oropharynx (OPSCC), including the tonsil/base of tongue region, with HPV status usually determined by expression of a surrogate marker p16 (INK4A).^4,5^ HPV^+ve^ and HPV^−ve^ tumours have significantly different molecular profiles and clinical behaviour, particularly with respect to invasion, metastasis and response to chemoradiotherapy.^6–8^ PET imaging is routinely used for identification, staging and follow up of HNSCC, however no studies have examined how HPV status affects the metabolic phenotypes of these tumours, which may in turn affect the clinical ability to detect tumours. There is also increasing evidence of fundamental links between a tumour’s metabolic phenotype, its interactions with the tumour microenvironment and clinical outcome,^9–12^ suggesting that specific metabolic pathways may promote tumour progression and represent therapeutic targets. Understanding the metabolic profiles of HNSCC cancer subtypes may therefore impact on the clinical management of this disease.

## Materials and methods

### Cell lines, culture and treatment

HNSCC-derived HPV^−ve^ cell lines SCC-25^11^ and UM-SCC89^12^ were cultured in standard medium consisting of Dulbecco’s modified Eagle’s medium (DMEM; Sigma Aldrich):Ham’s F12 (1:1; BioWhittaker), containing 10% (v/v) foetal bovine serum (FBS) and 2 mM L-glutamine. Two immortalised cell lines derived from the upper aerodigestive tract from suspected HPV^+ve^ tumours, UD-SCC-2 and UPCI:SCC90,^13,14^ were kindly supplied by Susanne M. Gollin (University of Pittsburgh, USA; shortened to SCC2 and SCC90 respectively in results). These cell lines, along with Detroit 562, an additional HNSCC cell line used in functional analyses, were cultured in 10% FBS supplemented DMEM. Polymerase chain reaction (PCR) was performed to confirm the HPV status of cell lines used in functional assays by the presence of E6/E7 oncoprotein RNA (Supplementary Figure 1). PCR was also utilised to perform testing for mycoplasma throughout experimentation. Cell counting for all functional assays was performed utilising a CASY counter (Innovatis AG, UK/Roche Diagnostics GMBH). AZD3965 (AstraZeneca, Waltham, MA) was reconstituted according to the manufacturer’s instructions to a working stock concentration of single use aliquots at 1 uM solution in DMSO. Recent publications^15^ and a kill curve for the compound on SCC-25 cells determined 10 nM inhibitor concentration as the functional dose for functional analysis (Supplementary Figure 2); cells were exposed to AZD3965 for 48 h prior to use in assays unless otherwise stated.

### Measurement of oxygen consumption rate (OCR) and extracellular acidification rates (ECAR)

OCR and ECAR measurements were performed using the XF96 Extracellular Flux analyzer (Seahorse Bioscience, North Billerica, MA) as described in Wu et al.^16^ Briefly, cells (2 × 10^4^/well) were plated in 80 µl of growth medium in a XF96 culture plate well, while the probes were immersed in XF Calibrant Solution (Seahorse Bioscience; lacking sodium bicarbonate and FCS) overnight at 37 °C in non-CO_2_ incubator 24 h prior to experimental run. Running medium for the assay was prepared using glucose-free DMEM supplemented with 10% serum, 2 mM L-glutamine (PAA) and 1 mM sodium pyruvate (PAA), and the pH was adjusted to 7.4 at 37 °C. Prior to performing the assay, drugs from mitochondrial and glycolysis stress kits (Seahorse Bioscience) were diluted in running medium and loaded into injection ports of the probe cartridge. Cells were washed twice with 200 µl of running medium, and the plate was placed in a non-CO_2_ 37 °C incubator whilst sensor cartridges were calibrated. OCR and ECAR were measured over a 2-minute period as absolute rates (pmoles/min and mpH/min respectively), followed by 3-min mixing and re-oxygenation of the media. Experimental conditions in each assay were performed with a minimum of triplicate wells. Glycolytic capacity was calculated using the equation: maximum rate measurement after oligomycin injection—last rate measurement before glucose injection, expressed as mpH/min. A standard Bradford assay (Bio-Rad Laboratories) analysis was performed on end-point wells to normalise to total protein count per well (ug/well).

### Western blot analysis

Cells were lysed in NP40 buffer (Biosource, Invitrogen, Paisley, UK). Samples containing equal amounts of protein were electrophoresed under reducing conditions in 8–10% SDS-PAGE gels. Protein was electroblotted to PVDF membranes (Amersham Biosciences, Buckinghamshire, UK). Blots were probed with antibodies against MCT1 (Santa Cruz Biotechnology). Horseradish peroxidase-conjugated anti-rat or anti-mouse (Dako) was used as secondary antibodies. Bound antibodies were detected with the enhanced chemiluminescence western blotting detection kit system (Amersham). Blots were probed for Total FAK (Millipore) as a loading control.

### Invasion assay

Cell invasion was analysed using Transwell® assays (8 μm pore size, polycarbonate membrane, Corning® Costar® Wiesbaden, Germany) as previously described.^17^ Briefly, a layer of Matrigel (BD Biosciences, San Diego, CA, USA) diluted 1:2 with DMEM was placed on top of each insert prior to seeding 5 × 10^5^ cells/200 µl in each chamber. A 72-h incubation at 37 °C was performed to allow invasion of cells to the lower chamber and counted using a CASY counter (Innovatis AG, UK/Roche Diagnostics GMBH).

### Organotypic culture

Organotypic cultures were prepared as previously described.^17^ A 1ml mixture comprising 3.5 volumes type I rat tail collagen (Merck Millipore), 3.5 volumes Matrigel (Becton-Dickinson), 1 volume 10x DMEM, 1 volume foetal calf serum and 1 volume of 10% DMEM together with 2.5 × 10^5^/ml HFFF2 cells was allowed to polymerise. A mixture of HNSCC cells (5 × 10^5^) and HFFF2 fibroblasts (25 × 10^4^) in a combined volume of 1ml of DMEM was then added dropwise onto the top of the gel. After 24 h incubation, the gels were raised onto nylon sheet coated stainless steel grids. After 7 days incubation at 37 °C the gels were bisected, fixed in formal-saline and processed to paraffin. Sections (4 μm) were stained for H&E ± pan-cytokeratin.

### Clonogenic survival assay

SCC-25 cells were treated with 10 nM AZD3965 or a DMSO control for 48 h prior to the start of the survival assay, trypsinised, counted and seeded in 6-well plates at 2000 cells/well. AZD3965/DMSO containing media was replaced after 24 h and then left in situ for 10 days until conclusion of the experiment.

To test radiosensitivity, the cell suspensions were exposed to a range of γ-ray doses in 2-Gy increments dose of γ-rays prior to plating at 2000 cells/well. Standard cell media was utilised for both control and AZD3965 pre-treated populations to homogenise post radiation exposure conditions.

At completion cells were fixed in a 3% crystal violet/10% formalin solution. Following collation of scanned images, the ‘ColonyArea’ plugin of Image J^18^ was utilised, allowing automated thresholding and analysis, to calculate both % area and intensity of colonies.

### RNA sequencing and data analysis

Single ended 35 bp read length RNA-Seq was performed with mRNA from 39 consecutively collected HNSCC tumour samples as described in Wood et al.^19^ (data available in Gene Expression Omnibus, accession number GSE72536). At the time of the present study, 35 patients in this cohort initially had sequencing data available for analysis. The quality of raw SE read data was assessed and reads of low quality were removed using PRINSEQ and reads with low complexity were remove using DUST, complexity scores above 7 were removed. SE reads were then mapped to the human genome (hg19) using TopHat (version 2.0.9) allowing no mismatches. Only reads uniquely aligned were considered for further counting using HTSeq-count (version 0.5.4), yielding read count values for a total of 23,368 RefSeq annotated genes. The raw counts were further processed in Bioconductor package EdgeR (version 3.4.2). Nonspecific filtering of count data was performed such that genes with less than 2 read counts per million in 25% of the samples were removed. The remaining 14565 genes (Supplementary Table 1A) were subject to normalisation using the trimmed mean of M-values (TMM) normalisation method to account for differences in library size between samples. The TMM normalised data was subject to differentially expressed gene (DEG) analysis using generalised linear model likelihood ratio test. A gene was considered significantly differentially expressed when the FDR adjusted, using Benjamini and Hochberg method, p-value was lower than 0.05 and absolute fold change greater than 2. To examine metabolism related genes, we collected ID codes for all metabolism-related sub-pathways from the KEGG PATHWAY database.^20^ MetabolicMine^21^ was then utilised to perform a query for all genes involved in the identified pathways. Non-human species results were excluded, and all gene symbols converted into ensemble IDs (Supplementary Table 2). All genes were matched to related IDs (3 genes lost in cross conversion (GALNTL2, GALNTL4, PTPLA)). The DEG results were then correlated with the metabolism related gene database.

### Gene ontology (GO) and pathway analysis

GO terms associated with biological processes and biological pathways that were significantly over-represented for DEGs (*q*-value < 0.05) were identified with ToppGene web tool^22^ and functional enrichment analysis was performed using ToppFun from the ToppGene suite. Statistical significance of different GO terms and pathways were estimated using hypergeometric testing with FDR adjustment for multiple testing using the Benjamini and Hochberg method. GO terms and functional pathways with FDR corrected *p*-value < 0.05 was considered significant. Significant GO terms were visualised in semantic similarity-based scatterplots generated by REVIGO.

### TMA production and immunohistochemistry

Immunohistochemical analysis was performed on a tissue microarray of consecutively-treated patients with oropharyngeal squamous cell carcinoma (OPSCC; *n* = 260 (231 with confirmed HPV status)) from University Hospital Southampton (2000-10), Poole NHS Foundation (2000-6) and Bart’s and the London NHS Trust (2000-6) as described previously^23^ (REC references 09/H0501/90 and 07/Q0405/1). Tumours classified as HPV^+ve^ were positive for both p16 immunohistochemistry and HPV ISH. Immunochemistry was performed using MCT1 antibody (Santa Cruz Biotechnology) and scored by two blinded investigators (KM/JF) as high (moderate/high strength staining) or low (absent/low strength staining).

### Systematic review

The following broad search strategy was utilised on Pubmed/Medline and Embase: ((FDG OR PET) AND (HPV OR papillomavirus)), with all fields included and including dates 1946-present. Titles and abstracts were scrutinised and full texts of relevant and related articles were retrieved to enable final selection. Only cases reporting SUV(max) scores were included. Case reports and commentaries were excluded. Only English language articles analysing the direct comparison between FDG-PET results in HPV^+ve^ and HPV^−ve^ HNSCC were included. Studies only evaluating other tumour types or without differentiation between viral and non-virally derived HNSCC were excluded. Reference lists of key articles were cross-referenced to identify additional articles. Where mean SUV(max) was not reported, indicated mean values were estimated from reported median, range and sample size.^24^ A mean difference approach was used for meta-analysis and a Forrest plot to examine effect created using Review Manager version 5.0 (RevMan).^25^

### Statistical analysis

For comparisons between experimental groups in functional assays, a Student’s *T*-test was used. For other datasets where normal distribution was not evident, non-parametric data analysis was performed (Mann–Whitney test for unpaired, Wilcoxen matched-pairs signed rank test; Prism v6 for Mac, Graphpad Software). Error bars show standard deviation (SD) unless otherwise stated. For TMA/database analysis, SPSS Statistics (v22 for Mac, IBM, NY) was used.

The primary endpoint for survival analysis was death from OPSCC (i.e., disease-specific survival). An OPSCC-specific survival time was measured from the date of diagnosis until date of death from OPSCC or date last seen alive. Kaplan–Meier survival curves were produced using clinicopathological patient data. Death from other causes or loss to follow up was marked as censored for analysis.

For all analyses, a p value of equal or less than 0.05 was considered to be significant.

## Results

### Transcriptional landscape of HNSCC cell metabolism

**Fig. 1:**
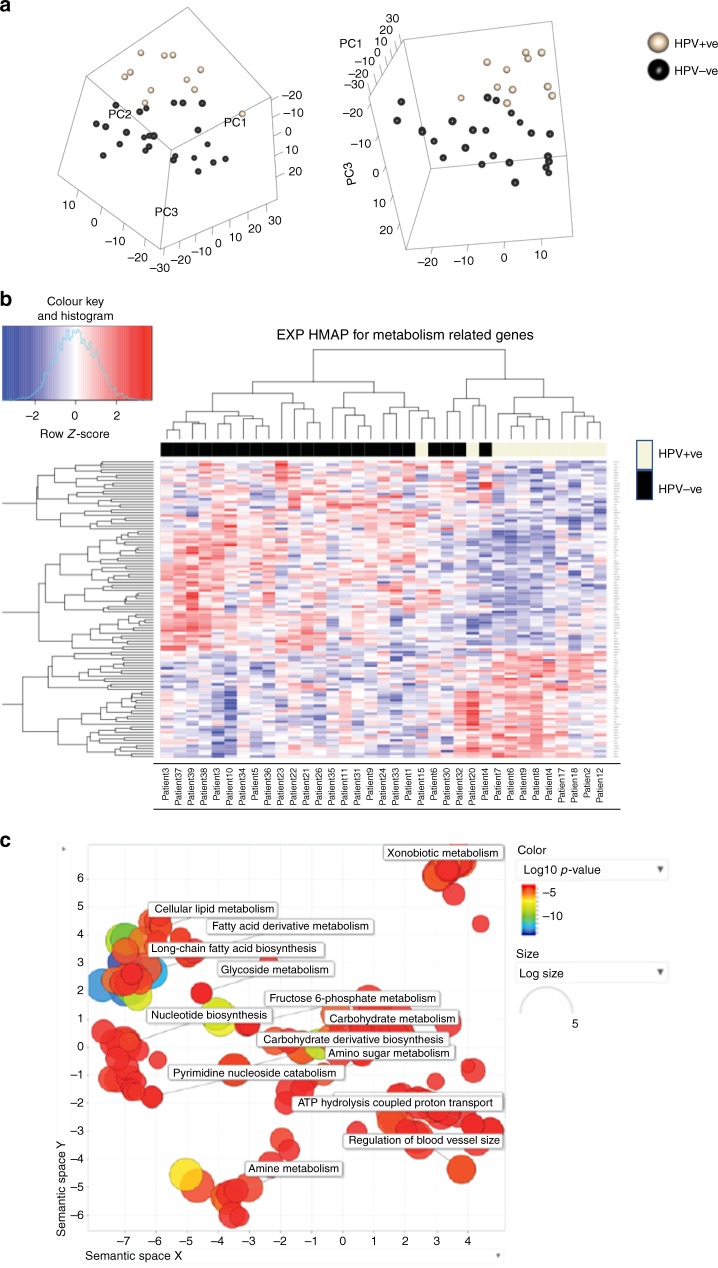
Analysis of RNA sequencing data from HPV^+ve^ (*n* = 11) and HPV^−ve^ (*n* = 24) HNSCC. **a** Unsupervised Clustering of 1205 metabolism-related genes with a Principal Component Analysis (PCA plot). **b** Genes involved in global metabolism pathways were extracted from KEGG and their expression correlated with the differentially expressed genes (DEG) in HPV^−ve^ and HPV^+ve^ tumours to produce a heatmap of 111 genes. **c** Gene ontology analysis of 111 common metabolism-related genes differentially expressed between HPV^+ve^ and HPV^−ve^ HNSCC. Analysis was performed in ToppGene and summarised as a scatterplot of cluster representative terms in REVIGO based on semantic similarity. Bubble colour indicates the user-provided p-value and bubble size indicates the frequency of the GO term in the underlying database (general terms produce larger bubbles). Clusters with the largest and most significant values have been highlighted with text descriptions

Current understanding of the relationship between HPV status and HNSCC metabolism is limited. 35 HNSCC tumours (11 HPV^+ve^, 24 HPV^−ve^) were analysed by RNA sequencing^19^ to produce a differentially expressed gene (DEG) list of 1587 genes based on viral status (Supplementary Table 1B). We correlated this list with a publicly available KEGG gene database of metabolism-related genes, and then performed unsupervised clustering analysis on this metabolism dataset using principal component analyses; this separated the tumour subgroups according to HPV status (Fig. 1a), confirmed on *t*-Distributed Stochastic Neighbour Embedding (Supplementary Figure 3). 111 metabolism-related genes were identified amongst the DEG between HPV^+ve^ and HPV^−ve^ tumours (Fig. 1b); DEGs by cluster are listed in Supplementary Figure 4.

To identify differences in specific metabolic pathways between HPV^+ve^ and HPV^−ve^ tumours, we performed gene ontology (GO) analysis on the 111 differentially expressed, metabolism-related genes and summarised results in REVIGO^26^ (Fig. 1c). The top GO terms that highlighted differences between HPV^+ve^ and HPV^−ve^ tumours included significant alterations in gene expression (identifying both upregulated and downregulated genes) associated with nucleotide biosynthesis and carbohydrate metabolism.

### Metabolic enzyme expression analysis

**Fig. 2:**
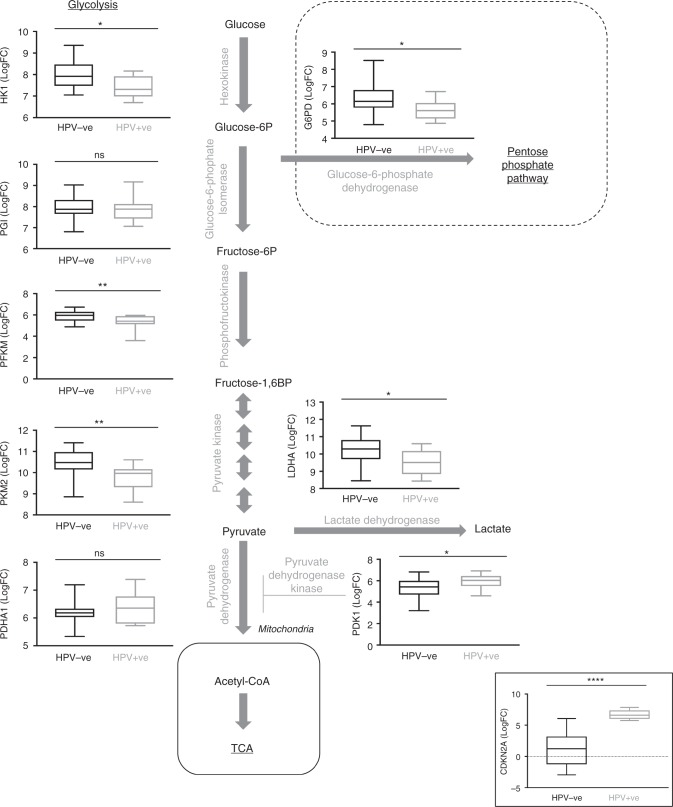
RNA expression panel of key metabolic genes comparing HPV^+ve^ (*n* = 11) with HPV^−ve^ (*n* = 25) HNSCC. CDKN2A (p16^INK4a^; lower right panel) is included as validation for determination of HPV positivity as clinically corroborated with IHC. Pooled fold change (logFC) for tumours compared with non-parametric Mann–Whitney test following graphical confirmation of non-normalised data distribution. Significantly increased expression levels in HPV^−ve^ tumours are observed for HK1 (*p* = 0.0106), PFKM (*p* = 0.0076), PKM2 (*p* = 0.0022), LDHA (*p* = 0.0146) and G6PD (*p* = 0.0178) whilst PDK1 alone was elevated in HPV-positive tumours (*p* = 0.0452). No difference between groups was observed for PGI (*p* = 0.4766) and PDHA1 (*p* = 0.6116). *ns* not significant, *p ≤ 0.05; **p < 0.01; ****p < 0.0001

Since gene ontology analysis revealed HPV-related differences in carbohydrate metabolism (Fig. 1c), we next examined glycolysis and related pathways in more detail, focusing on gene expression of eight key enzymes (Fig. 2). Four enzymes from glycolysis were included: hexokinase (HK1), phosphoglucose isomerase (PGI), phosphofructokinase (PFKM), pyruvate kinase (PKM2, the major isoform in tumours) and lactate dehydrogenase (LDHA). Other enzymes included glucose-6-phosphate dehydrogenase (G6PD) from the pentose phosphate pathway, pyruvate dehydrogenase (PDHA1) and its kinase (PDK1) controlling entry into the TCA cycle. This was supplemented by analysis of additional genes associated with the pentose phosphate pathway (Supplementary Figure 5a) and glucose metabolism (Supplementary Figure 5b).^27^ We found that there was upregulation of glycolysis genes (HK1, PFKM, PKM2, LDHA significantly upregulated) in HPV^−ve^ HNSCC, which also expressed lower levels of the negative regulator, PDK1 (Fig. 2). Differences in the pentose phosphate pathway were less clear; while expression of G6PD was upregulated in HPV^−ve^ tumours, other pentose pathway genes were not differentially expressed (Supplementary Figure 5a). Analysis of the DEG list in the cellular protein domain also revealed significant enrichment of mitochondrial matrix proteins (*p* = 6.962 × 10^−6^; Supplementary Figure 6). These data suggest a more globally metabolically active signature in HPV^−ve^ tumours.

### Real time extracellular flux analysis of HNSCC cell lines

**Fig. 3:**
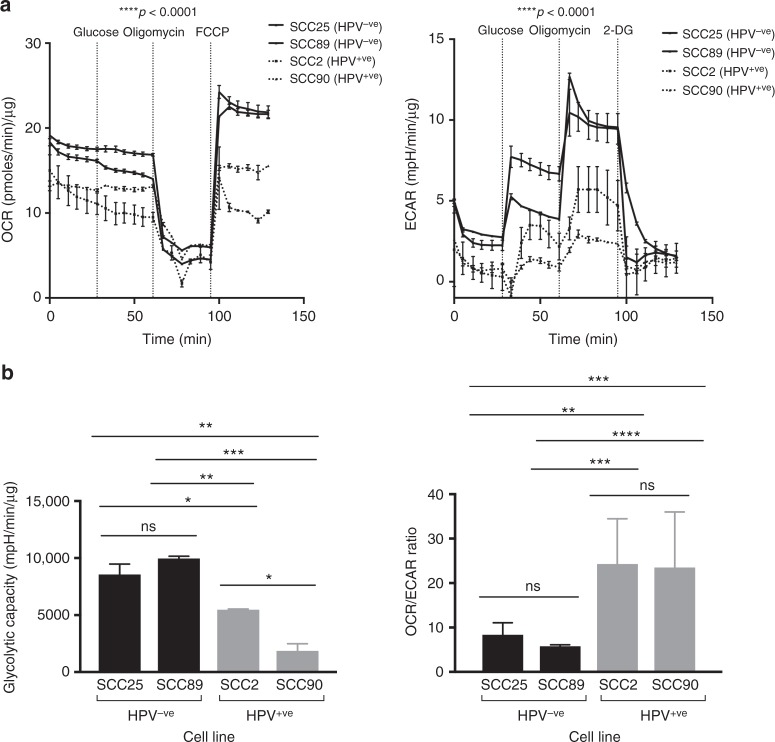
Bioenergetic profile of HPV^+ve^ (UD-SCC-2 [SCC2] and UPCI:SCC90 [SCC90]) and HPV^−ve^ (SCC-25 [SCC25], UM-SCC-89 [SCC89]) cell lines. **a** The oxygen consumption rate (OCR; left panel) and extracellular acidification rate (ECAR; right panel) were measured using an extracellular flux analyzer (Seahorse Bioscience) to estimate glycolysis and mitochondrial respiration respectively. Port injections at indicated times included glucose (5mmol/l), the ATP synthase inhibitor oligomycin A and finally FCCP to uncouple mitochondria or 2-DG to cease glycolysis. HPV^−ve^ cell lines demonstrated significantly globally elevated OXPHOS and glycolytic rates (*p* < 0.0001). **b** Histograms showing significantly increased glycolytic capacity in HPV-ve vs HPV+ve cell lines. A significantly reduced OCR/ECAR ratio in HPV^−ve^ compared to HPV^+ve^ cell lines indicates the latter are more reliant on oxidative phosphorylation for ATP requirements than glycolysis (ANOVA, Tukey's multiple comparisons, *p* < 0.01 for all comparisons of HPV^-ve^ vs HPV^+ve^ cell lines). Error bars represent SD and experiments (*n* = 2) were performed with a minimum of triplicate well repeats. *ns* not significant, *p ≤ 0.05; **p < 0.01; **p < 0.001; and ****p < 0.0001

To corroborate in vivo findings, we next examined the metabolic phenotype of HNSCC cells in vitro, focussing specifically on glycolysis and oxidative phosphorylation (OXPHOS). Four HNSCC cell lines, two HPV^−ve^ (SCC-25, UM-SCC-89) and two HPV^+ve^ (UD-SCC-2, UPCI:SCC90) were analysed using a Seahorse XF96 Extracellular Flux Analyzer (Seahorse Bioscience, North Billerica, MA) (Fig. 3), measuring oxygen consumption rate (OCR) and extracellular acidification rate (ECAR), which were used to extrapolate cell mitochondrial respiratory function and glycolytic function respectively. The HPV^−ve^ cell lines had significantly higher basal ECAR and OCR compared to the HPV^+ve^ cell lines (*p* < 0.0001; Fig. 3a). The difference between maximal and basal ECAR is considered the glycolytic reserve capacity of cells; this revealed that HPV^−ve^ cells have potential for much higher glycolytic rates than HPV^+ve^ cells (Fig. 3b, left panel). The OCR/ECAR ratio showed that UD-SCC-2 and UPCI:SCC90 were more reliant on mitochondrial respiration for their energy requirements (Fig. 3b, right panel). Notably, despite their increased reliance on glycolysis, the respiratory capacity of SCC-25 and UM-SCC-89 was also greater than the HPV^+ve^ lines, indicating a greater potential for ATP production and theoretically allowing cells to switch between OXPHOS and glycolysis to satisfy greater bioenergy demands depending on various tumour-related factors such as blood supply, hypoxia and the surrounding microenvironment. Both HPV^+ve^ cell lines appeared to be operating closer to their maximal respiratory capacity under standard cell culture conditions.

### Correlation of glycolytic phenotype with FDG PET imaging

The distinct in vivo and in vitro metabolic profiles between HPV^+ve^ and HPV^−ve^ HNSCC prompted us to investigate whether these differences were apparent on FDG-PET imaging. Prior research has been limited by HPV^−ve^ sample size due to the emerging dominance of HPV^+ve^ tumours in the oropharynx, and the previous relatively confined use of PET imaging to this anatomical site. We therefore performed a systematic review of the literature and produced a virtual data-series from several comparative studies, for analysis using SUV(max), which has been previously proposed as the most accurate and reproducible SUV measure.^28^

**Fig. 4:**
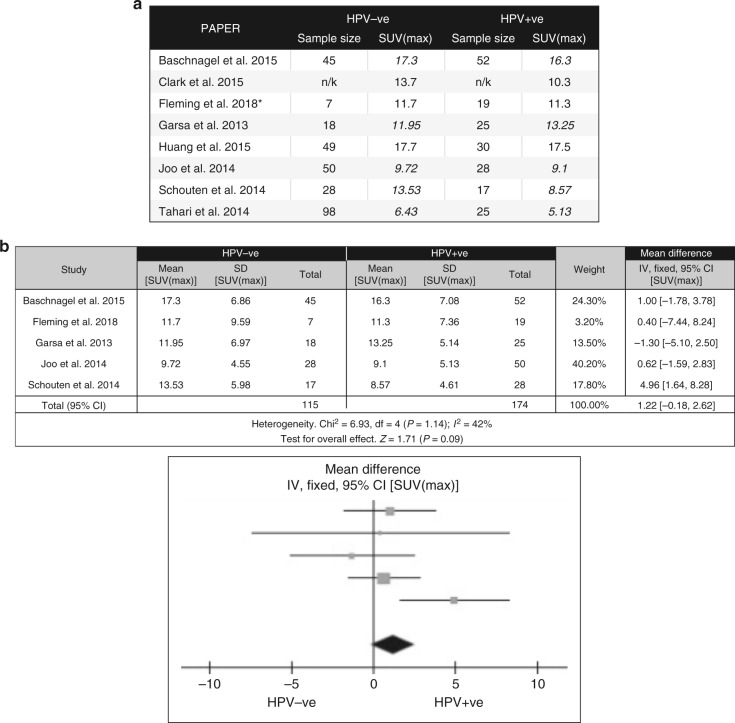
Comparison of combined FDG-PET derived SUV(max) scores for HPV^−ve^ and HPV^+ve^ tumours from a meta-analysis of the available literature (1946–present). **a** Table of collated mean SUV(max) scores alongside sample numbers for all relevant studies (* = current study). Where mean SUV(max) was not reported, indicated mean values (italics) were estimated from reported median, range and sample size^25^ (*n/k* not known). **b** Table and Forest plot of comparison of SUV(max) for HPV^−ve^ vs HPV^+ve^ patients (mean difference, fixed effect analysis). Difference in means was +1.22 (−0.18–2.62 [95% CI]), indicating a strong trend for higher SUV(max) scores in HPV^−ve^ disease although no overall significance difference was proven

We identified a total of 305 studies; following de-duplication, 243 studies were screened by title ± abstract. 229 were excluded following initial screening. Full articles for the remaining 14 papers were reviewed and from this, 7 studies were excluded due to not fitting study aims (4) or insufficient data (3) (Supplementary Figure 7). The data from the remaining 7 studies were combined with our own database from two tertiary centres (N = 26; Age range 42–87 years; Fig. 4a). Our results indicated a mean SUV(max) score of 11.7 in the HPV^−ve^ cohort (*n* = 7, SD = 9.59) and 11.3 in the HPV^+ve^ (*n* = 19, SD = 7.36). A meta-analysis was performed on collated results, utilising a mean difference approach (Fig. 4b). Summary statistics showed there to be moderate heterogeneity between studies (*I*^2^ statistic = 0.42), with no overall difference between SUV(max) scores based on HPV status, although there was a strong trend for higher SUV(max) scores in the HPV^−ve^ populations (*Z* statistic = 1.71 (*p* = 0.09; mean difference = 1.22 (95% CI −0.18–2.62). Although the trend for higher SUV(max) scores in HPV^−ve^ tumours is consistent with our in vitro findings, the result suggests that, at a single timepoint, SUV(max) does not accurately distinguish between HPV^+ve^ and HPV^-ve^ HNSCC.

### Application of metabolic analysis to identify a new therapeutic target

**Fig. 5:**
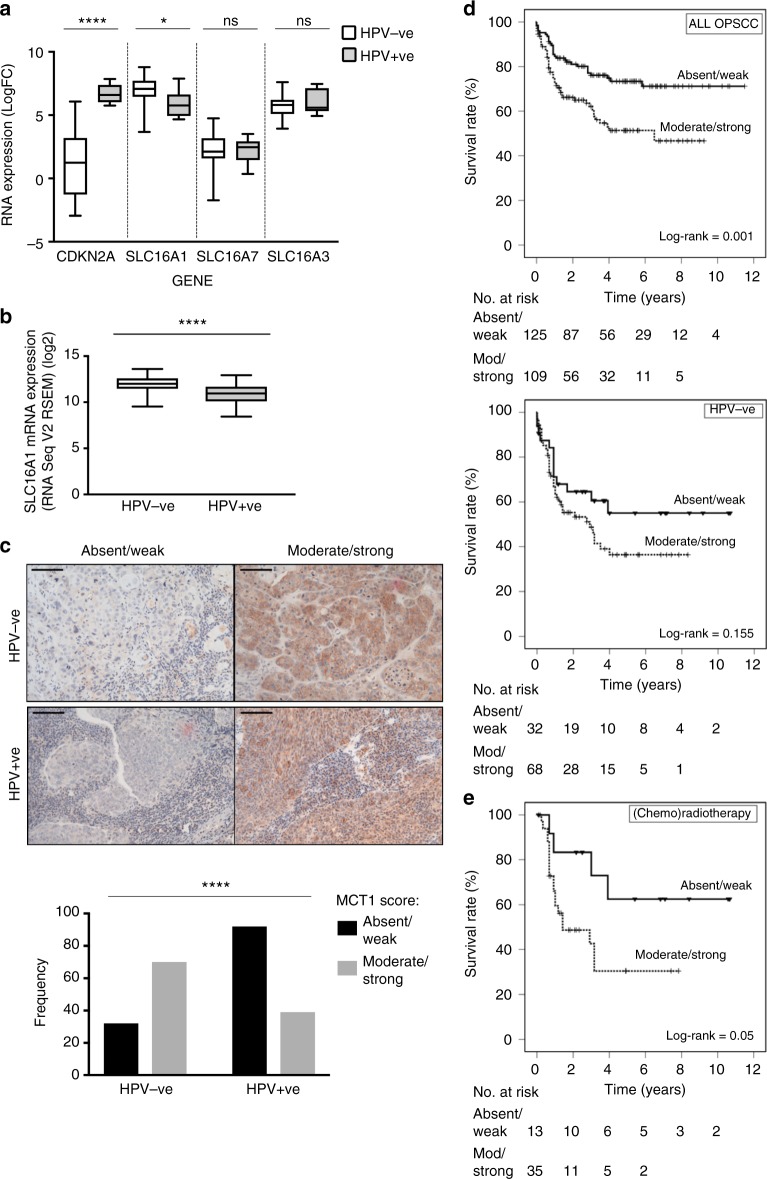
In vivo expression and clinical significance of MCT1 in HNSCC. **a** Analysis of RNA sequencing data for expression of MCT1 (gene SLC16A1), MCT2 (SLC16A7) and MCT4 (SLC16A3) in HPV^+ve^ (*n* = 11) vs HPV^−ve^ (*n* = 25) HNSCC. CDKN2A (p16^INK4a^) is included as validation for HPV positivity corroborating immunochemistry. Read counts compared with non-parametric Mann–Whitney test following graphical confirmation of non-normalised data distribution. Significantly increased expression levels in HPV^-ve^ tumours are observed for MCT1 (*p* = 0.0012). **b** TCGA HNSCC dataset (*n* = 279; 243 HPV^−ve^, 36 HPV^+ve^) was interrogated for MCT1 (SLC16A1) RNA expression and log2 values are plotted against viral status, confirming increased expression in HPV^−ve^ tumours (*p* < 0.0001). **c** Representative immunohistochemical staining showing examples of MCT1 expression levels in HPV-ve and HPV+ve HNSCC. Scale bar = 100 microns. MCT1 expression overall was significantly higher in HPV^−ve^ tumours (Chi-squared, *χ*^2^ < 0.0001). **d** Kaplan-Meier disease-specific survival curves for all oropharyngeal SCC (OPSCC; top) and HPV^−ve^ OPSCC (middle) based on MCT1 expression scored on immunohistochemistry as low (absent/weak) or high (moderate/high). A significant correlation was evident between MCT1 expression and disease specific survival in OPSCC (log-rank = 0.001). **e** Kaplan–Meier in HPV^−ve^ patients treated with (chemo)radiation as primary modality demonstrating significant disease specific survival association in this cohort (log-rank = 0.05). Patients receiving neoadjuvant treatment, post-operative radiotherapy only or palliative doses were excluded. *ns* not significant, *p ≤  0.05 and ****p < 0.0001

The distinct metabolic profiles of HPV^+ve^ and HPV^−ve^ HNSCC raises the possibility that these differences may play a role in the differing patient survival and response to therapy in these tumour subgroups, as well as offering potential therapeutic targets. For example, in glycolytically-active HPV^−ve^ HNSCC cells, lactate homoeostasis is likely to play a significant role in cell survival, a process requiring specialised monocarboxylic acid transporters: MCT1-4.^29,30^ Although these transporters facilitate metabolic shuttles, they were not included in the public global metabolism related gene list used for earlier comparison (Supplementary table 2) and we therefore directly examined the RNA sequencing data for expression of MCT1, MCT2 and MCT4 (MCT3 was not included due to its restricted expression to retina/choroid plexus epithelium). MCT2 (SLC16A7, *p* = 0.565) and MCT4 (SLC16A3, *p* = 0.438) showed no differential expression between HPV^−ve^ and HPV^+ve^ HNSCC; MCT1 expression (SCL16A1) however, was significantly elevated in HPV^−ve^ tumours (*p* = 0.012), suggesting a potential tumour subtype-specific, metabolic target (Fig. 5a). Analysis of the TCGA HNSCC dataset (*n* = 279)^31^ confirmed higher expression of MCT1 in HPV^-ve^ tumour samples (Fig. 5b). Immunochemistry analysis of MCT1 expression in a large cohort of HNSCC patients (HPV^−ve^ *n* = 100; HPV^+ve^ *n* = 131)^23^ also showed significantly stronger MCT1 expression in HPV^-ve^ tumours (Fig. 5c; *p* < 0.0001; Mann-Whitney U test), correlating weakly with Glut1 expression (Spearman’s Rho = 0.228 *p* = 0.001) and tumour cohesion (pattern of invasion; Spearman’s Rho = 0.232 *p* = 0.001), but not with tumour grade, perineural, lymphatic or vascular spread. (Supplementary Figure 8a–c). MCT1 expression was negatively associated with disease-specific survival in all oropharyngeal HNSCC (Fig. 5d, upper panel; log-rank = 0.001;), but showed only a non-significant trend when subcategorised according to HPV status, more apparent in HPV^-ve^ tumours (Fig. 5d, middle panel, log-rank = 0.155; HPV^+ve^ HNSCC, Supplementary Figure 8d, left panel, log-rank = 0.706). Notably however, treatment sub-group survival analysis in HPV^−ve^ tumours showed that MCT1 expression had a significant negative association with survival in patients treated with primary (chemo)radiotherapy (Fig. 5e; log-rank = 0.05) compared with those treated surgically (Supplementary Figure 8d, right panel, log-rank = 0.668).

### Sensitivity of HNSCC cell lines to AZD3965, a novel MCT1 inhibitor

**Fig. 6:**
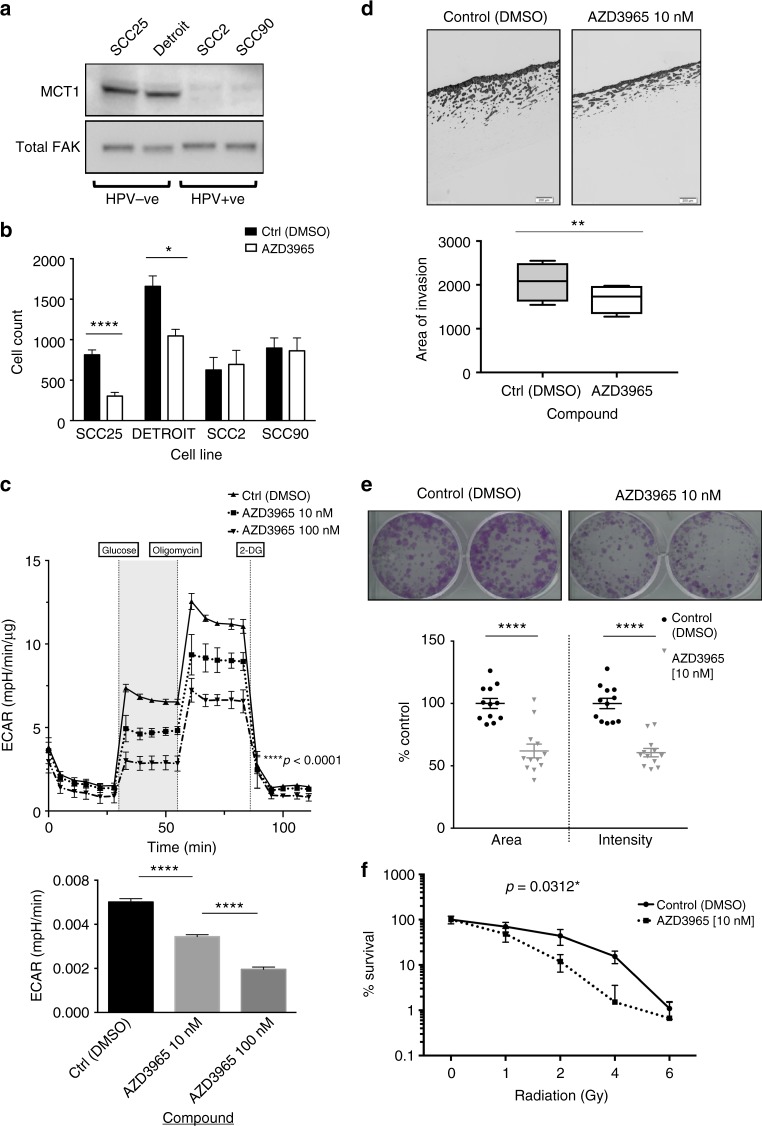
Functional analysis of HNSCC cell lines following MCT1 inhibition using AZD3965. Unless otherwise stated 10 nM compound was used in functional analyses with 48 h of incubation prior to cell assay. **a** Panel of HPV^-ve^ (SCC-25 [SCC25], Detroit 562 [Detroit]) and HPV^+ve^ cell lines (UD-SCC-2 [SCC2] and UPCI:SCC90 [SCC90]) were analysed for MCT1 expression by Western blotting. Total FAK was used as a loading control. **b** Correlative invasion assays in this cell panel were performed (*n* = 3; representative result shown). MCT1 inhibition suppressed invasion in HPV-negative cell lines only. **c** Bioenergetic profile of HPV-negative SCC-25 cell line following AZD3965 treatment; representative data is shown following treatment with two AZD3965 concentrations (10 nM, 100 nM; *n* = 2, minimum of triplicate wells). Port injections included glucose (5 mmol/l), the ATP synthase inhibitor oligomycin A and the glycolysis inhibitor 2-DG. Shaded area demonstrates differential glycolytic response to glucose injection highlighting the dose-sensitive response to MCT1 inhibition. Lower panel shows glycolytic rates for each cell line (maximal glycolysis after glucose addition – basal glycolysis) showing a significant dose-effect of MCT1 inhibition on suppressing glycolysis in SCC-25 cells (Friedman test; *p* < 0.001). **d** Organotypic cultures of SCC-25 cells following AZD3965 or vehicle treatment. Representative images are shown; scale bars = 200 um. Quantification of area of invasion (lower panel) showed that MCT1 inhibition significantly reduced invasion (**p < 0.01; Paired *T*-test; *n* = 3). **e** Clonogenic assays showing effect of MCT1 inhibition on SCC-25 cells. Results are presented relative to % control well. MCT1 inhibition produced a significant reduction in both colony area and intensity in SCC-25 (*p* < 0.0001). Representative images are shown and analysis performed utilising ColonyArea plugin on Image J.^54^ **f** Radiosensitisation of SCC-25 cells following MCT1 inhibition. Clonogenic assays are shown for control and MCT1-inhibited SCC-25 following exposure to 2Gy increment doses of radiation at 48 h with collated results shown (*n* = 2). Well area analysis was performed utilising ColonyArea plugin on Image J^54^ and final colony area results were converted to % of the non-irradiated well area for the control and AZD3965-treated cells separately to enable direct comparison (represented on log scale). *p ≤0.05; **p < 0.01; ****p < 0.0001

Targeting lactate balance has previously been suggested as a potential therapeutic option to target cancer cells.^30^ We therefore tested the effect of inhibiting MCT1 on HNSCC cell function in vitro, using AZD3965 (AstraZeneca, Waltham, MA), a selective inhibitor of MCT1,^15^ which is in early-phase clinical testing. We first confirmed MCT1 expression in a panel of HPV^-ve^ (SCC-25, Detroit 562) and HPV^+ve^ (UD-SCC-2 and UPCI:SCC90) cell lines (Fig. 6a). Given the correlation between MCT1 and tumour cohesion in the OPSCC patient cohort, we initially tested the effect of AZD3965 on cell invasion in Transwell invasion assays; only invasion of HPV^−ve^ cells was significantly reduced by MCT1 inhibition (Fig. 6b; *p* < 0.0001 and *p* < 0.05, SCC-25 and Detroit 562 respectively). Since highest expression of MCT1 was seen in SCC-25 cells, we analysed the effect of AZD3965 in more detail, using this cell line as representative of our target population of HPV^−ve^ HNSCC. Initially we performed extracellular flux analysis to analyse real-time metabolic outputs, following MCT1 inhibition. AZD3965 produced a dose-dependent reduction of glycolytic rate as well as glycolytic capacity (Fig. 6c). This was mirrored by an increase in mitochondrial respiration (Supplementary Figure 9). Organotypic assays were used to study invasion in a more physiological context, and similar to Transwell assays, showed a reduction following AZD3965 treatment (*p* < 0.01; Fig. 6d). In clonogenic survival assays, inhibition of MCT1 activity resulted in an approximately 50% reduction in colony survival and intensity (incorporating cell number) (Fig. 6e; *P* < 0.0001). Since the negative survival impact of MCT1 expression was most evident in patients treated with chemoradiotherapy, we hypothesised that AZD3965 may make SCC-25 cells more radiosensitive. SCC-25 cells were treated with AZD3965 for 48 h, and then exposed to incremental *γ*-rays doses up to a maximum of 6-Gy. AZD3965-treated cells were significantly more sensitive to irradiation (Fig. 6f; *p* = 0.0312).

## Discussion

HNSCC has high rates of glucose uptake,^32^ and FDG uptake in PET imaging reflects this,^33^ however, it is not clear whether metabolic differences exist between HPV^+ve^ and HPV^-ve^ HNSCC, tumour subgroups that show very different clinical behaviours. We found that HPV^−ve^ tumours have a more glycolytic phenotype compared to their HPV^+ve^ counterparts, but also have a globally altered metabolic profile consistent with the greater requirements for macromolecule production, as well as alterations in other metabolic and biosynthetic pathways. In keeping with recent advances in diverse metabolic targeting (reviewed by Luengo et al.^34^), RNA sequencing data showed enrichment in HPV^−ve^ tumours of additional metabolic pathways including those involved in fatty acid, lipid and nucleotide biosynthesis. Understanding the wider metabolic features of HPV^−ve^ tumours, and how this may affect response to treatment may enable us to develop treatment combinations that exploit metabolic dependence.

Findings of increased expression of glycolytic genes in HPV^−ve^ tumours  were supported by extracellular flux analysis confirming a higher rate of glycolysis and glycolytic capacity in HPV^−ve^ cell lines, in keeping with the requirement for redox balance in the presence of rapid cell division. It may also explain why HPV^−ve^ cell lines showed a greater glycolytic capacity in vitro, with their full glycolytic potential moderated by elevated PKM2 to allow for intermediate shuttling for nucleotide and NADPH production. Our finding of concurrent higher oxygen consumption rates in HPV^-ve^ cell lines is consistent with other studies noting that mitochondrial function is not impaired in the majority of cancer cells.^35,36^ The reason for differences in glycolysis between HPV^−ve^ and HPV^+ve^ HNSCC is uncertain; the three rate limiting reactions in glycolysis are catalysed by hexokinase, phosphofructokinase and pyruvate kinase, and all are relatively upregulated in HPV^−ve^ HNSCC. Recent studies show that TP53, a negative regulator of glycolysis, is mutated in around 75–85% of HPV-ve HNSCC.^31,37–39^

Hypoxia inducible factor 1 alpha (HIF1a) is also a potent promoter of glycolysis which is commonly expressed in HNSCC, being positively regulated by hypoxia and other factors including oncogenic signalling and reactive oxygen species.^40^

Ultimately the classification of metabolic phenotype in HNSCC may have important relevance in understanding the biology and clinical behaviour of the disease subgroups, indicating that glycolytic HPV^−ve^ tumours have a biosynthetic advantage over their HPV^+ve^ counterparts.^41,42^ Altered tumour metabolism may also provide opportunities for therapeutic targeting of specific metabolism-related pathways. MCT1 expression is differentially upregulated in HPV^−ve^ tumours and significantly associated with poor survival in OPSCC patients, notably in those with HPV^−ve^ disease treated with chemoradiotherapy. Similar correlation of MCT1 expression with poor prognosis has been reported in tumours of the breast,^43^ ovary,^44^ stomach^45^ and colon.^46^ Functional flux analysis of response to MCT1 inhibition indicated a shift in the metabolism profile of HNSCC cell lines from glycolysis to oxidative phosphorylation, resulting in suppressed tumour invasion and colony formation. Although previously considered a lactate importer, MCT1 has demonstrated bi-directionality^47^ which may help to explain the variable effects on cell metabolism produced by MCT1 inhibition reported in the literature. For example, in various cancer cell lines Bola et al.^48^ demonstrated that AZD3965 resulted in an increase in glycolysis and glycolytic enzymes whereas, similar to the present study, Doherty et al.^49^ found that MCT1 inhibition rapidly abrogated glycolytic function. The presumed reduction in intracellular pH following reduction of lactate efflux measured helps explain the negative cell survival effects on HNSCC cell lines following MCT1 inhibition observed in clonogenic assays. Areas of low pH in the tumour microenvironment have been demonstrated to correspond with cancer cell migration and invasion,^50,51^ therefore the reduction in lactate efflux with the MCT1 inhibitor may also explain the functional effects on cell migration and invasion. However, while these data suggest that MCT1 targeted therapy could be of potential benefit to patients with HPV^−ve^ tumours, further pre-clinical modelling is required to translate these finding.

FDG-PET is now a standard-of-care investigation in the diagnosis of primary and metastatic HNSCC, as well as being used for post-treatment surveillance. We found that SUV(max) alone cannot distinguish HPV status in primary tumours, especially given the range of reported metrics and variability of imaging parameters between centres. Standardised uptake value (SUV) measurements have been shown to be reproducible and have both diagnostic and prognostic value in HNSCC^52^ but with these imaging metrics alone, there have been conflicting reports in the literature about the significance of HPV status to glycolytic phenotype.^52,53^ It is possible that dynamic PET imaging over time could pick up differences in the rate of glucose uptake between HPV^-ve^ and HPV^+ve^ tumours. However, imaging at a single, static and relatively late time point, as currently occurs in clinical imaging protocols, does not distinguish between tumour subgroups.

In summary, we show that there are significant metabolic differences between HPV^−^^ve^ and HPV^+ve^ HNSCC; whilst this does not impact on the ability to clinically detect these subgroups using PET imaging, our data adds to the growing evidence that targeting glycolytic pathways may have therapeutic value in HPV^−ve^ disease.

## Supplementary information

Signed letter approving additional author

Supplemental Figure 1

Supplemental Figure 2

Supplemental Figure 3

Supplemental Figure 4

Supplemental Figure 5

Supplemental Figure 6

Supplemental Figure 7

Supplemental Figure 8

Supplemental Figure 9

Supplementary Table 1

Supplementary Table 2

Signed author letter

## Data Availability

Gene Expression Omnibus (GEO), accession number GSE72536
