# Supplementary figures and images for: HPV, tumour metabolism and novel target identification in head and neck squamous cell carcinoma
Source: Br J Cancer. 2019 Jan 17;120(3):356–67. doi: 10.1038/s41416-018-0364-7 (PMC6353968; doi:10.1038/s41416-018-0364-7)

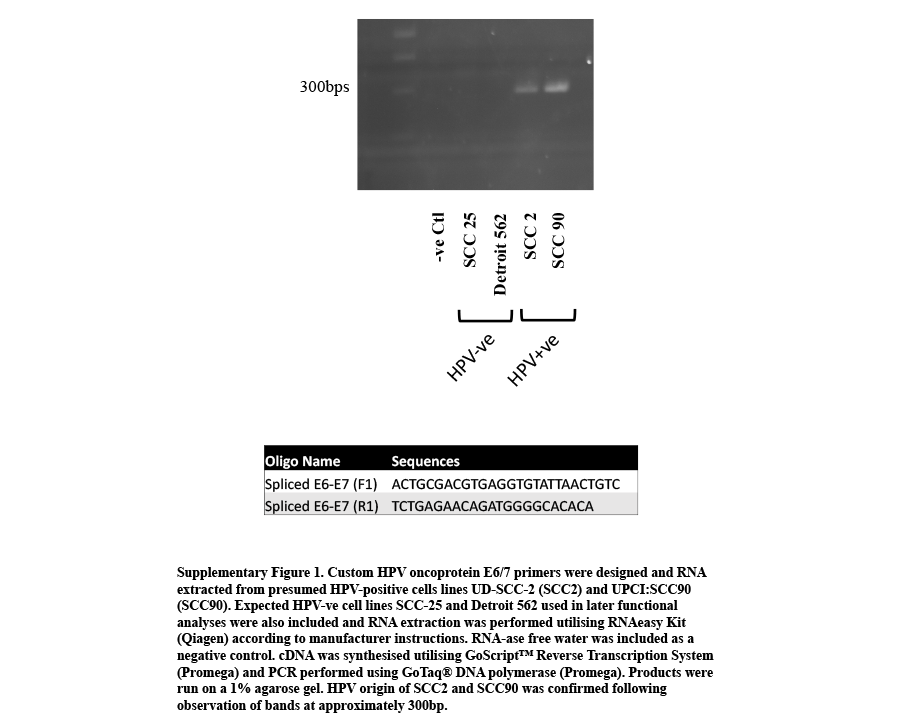

Supplement: Supplementary file 2 — Supplemental Figure 1 [file 41416_2018_364_MOESM2_ESM.tif]

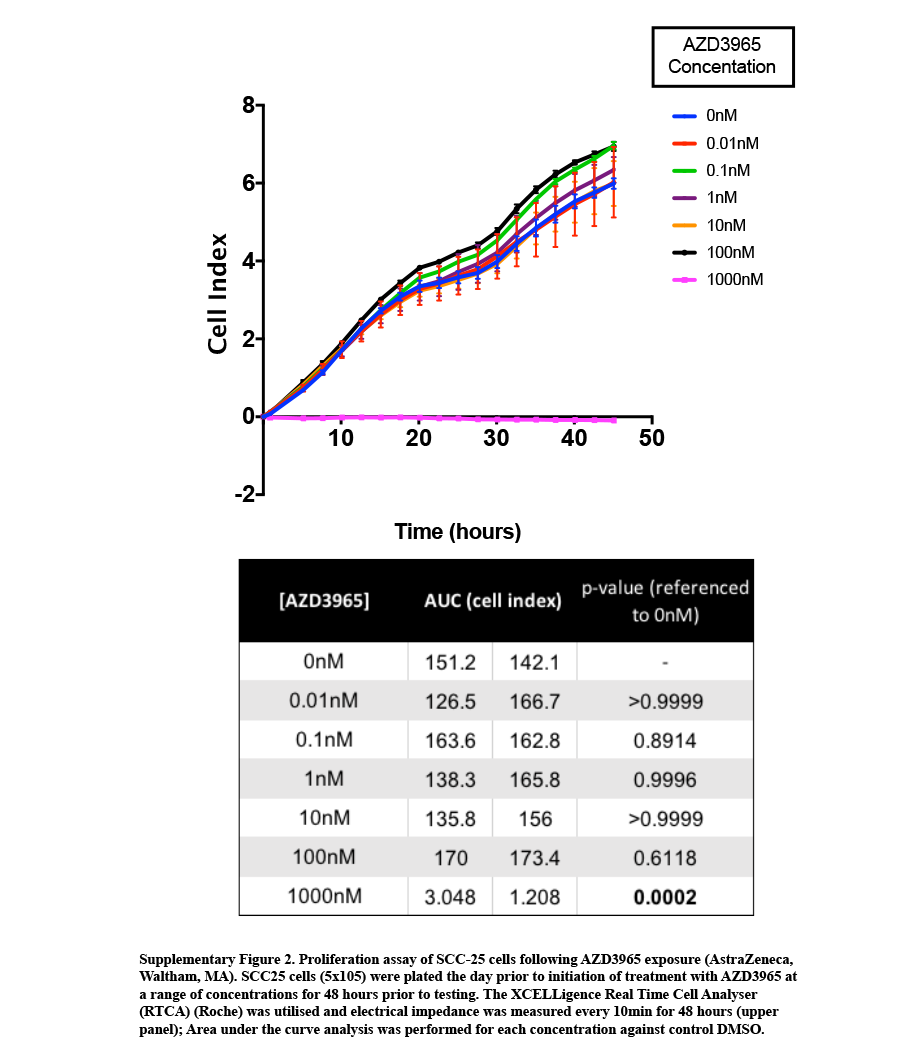

Supplement: Supplementary file 3 — Supplemental Figure 2 [file 41416_2018_364_MOESM3_ESM.tif]

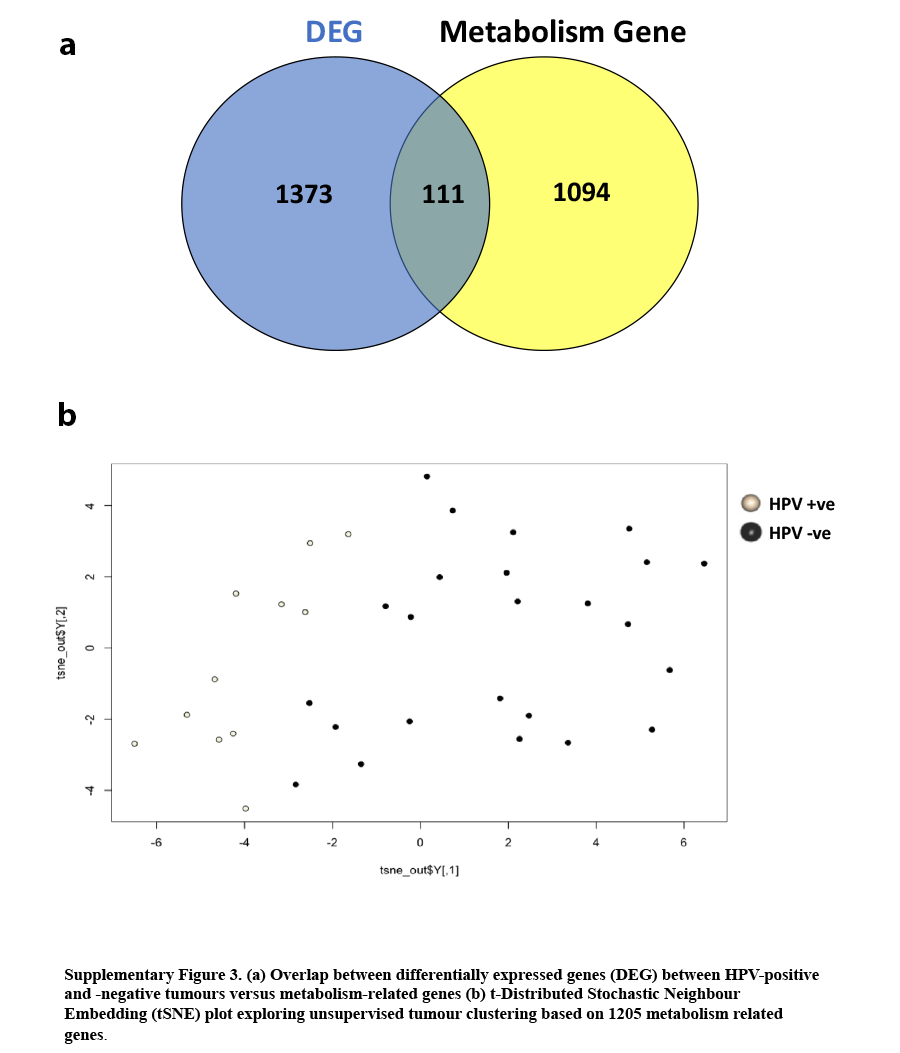

Supplement: Supplementary file 4 — Supplemental Figure 3 [file 41416_2018_364_MOESM4_ESM.tif]

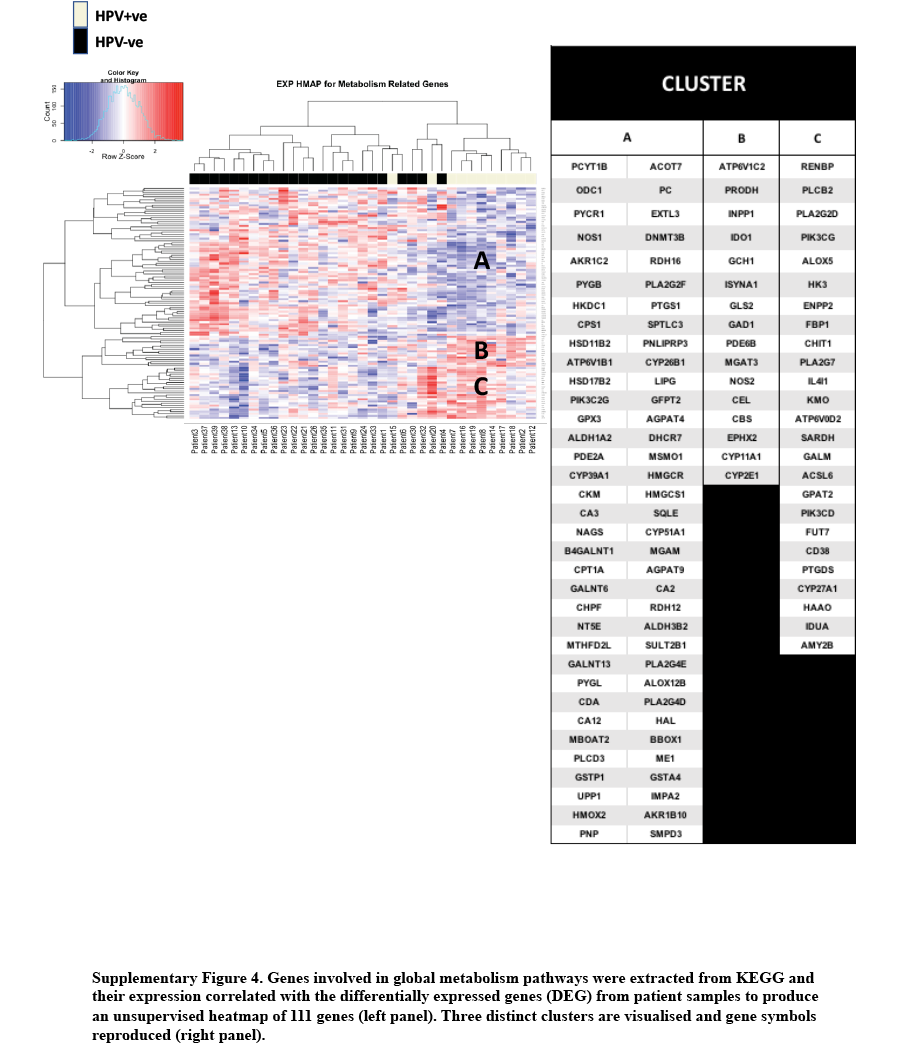

Supplement: Supplementary file 5 — Supplemental Figure 4 [file 41416_2018_364_MOESM5_ESM.tif]

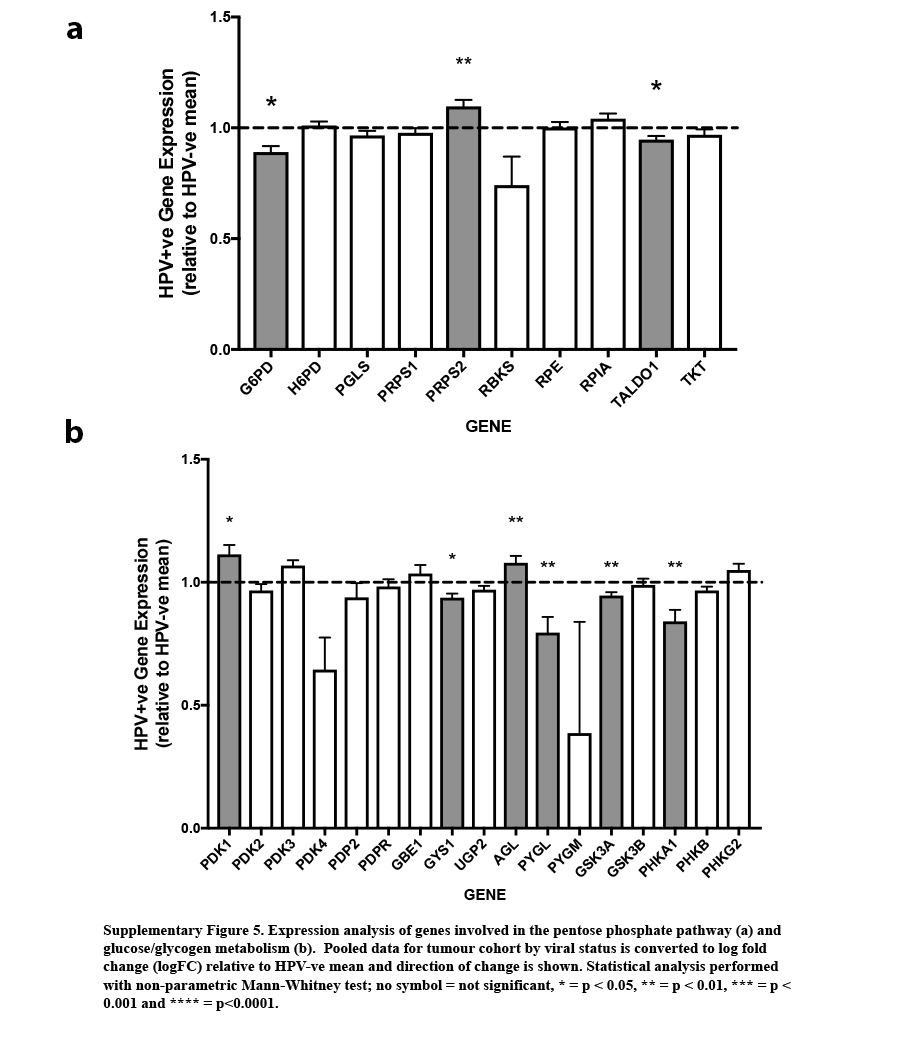

Supplement: Supplementary file 6 — Supplemental Figure 5 [file 41416_2018_364_MOESM6_ESM.tif]

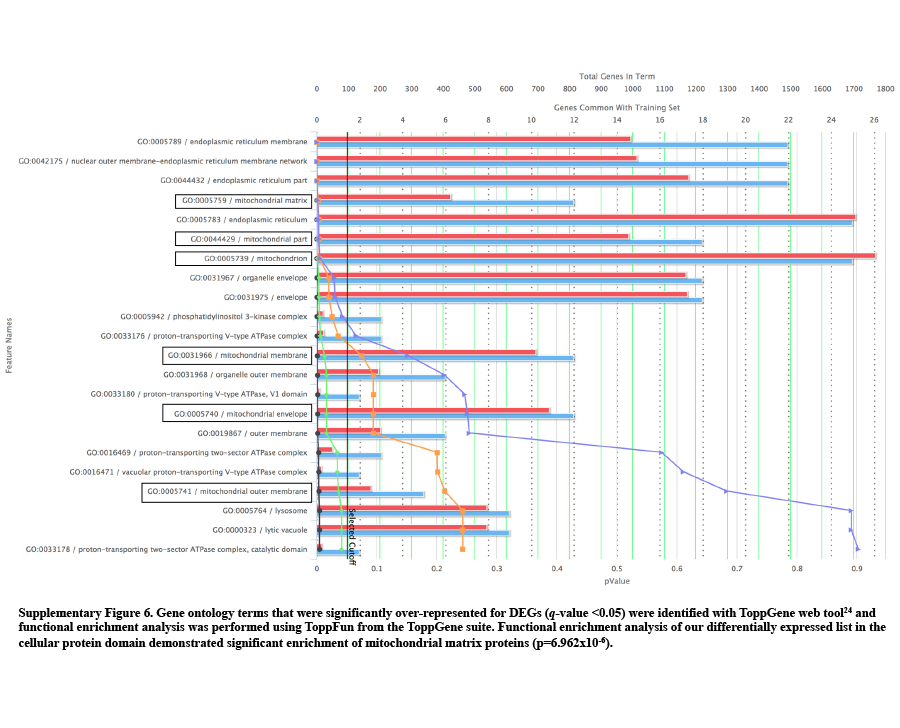

Supplement: Supplementary file 7 — Supplemental Figure 6 [file 41416_2018_364_MOESM7_ESM.tif]

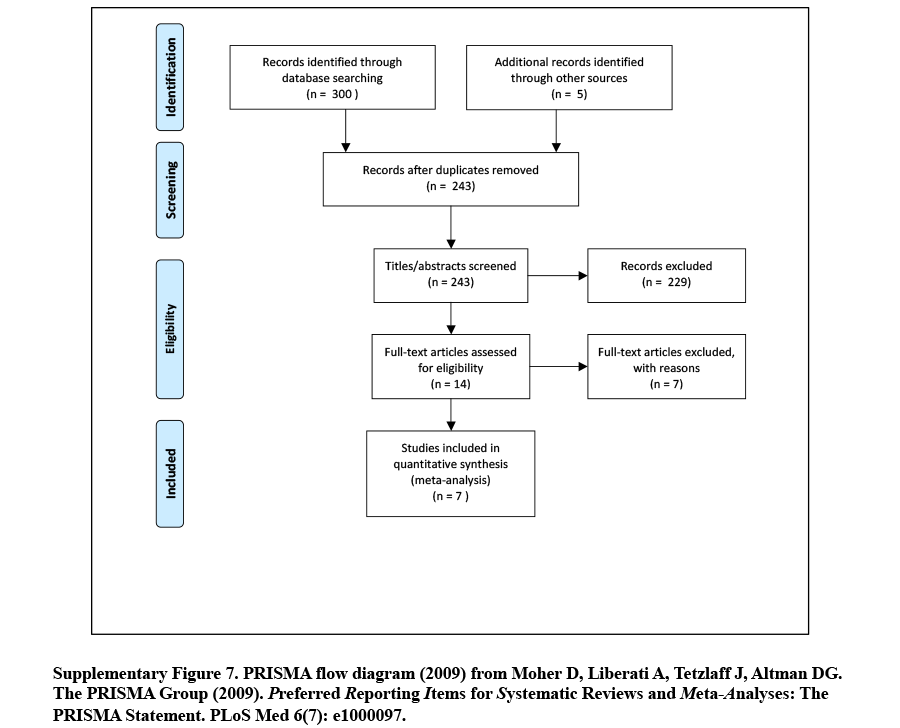

Supplement: Supplementary file 8 — Supplemental Figure 7 [file 41416_2018_364_MOESM8_ESM.tif]

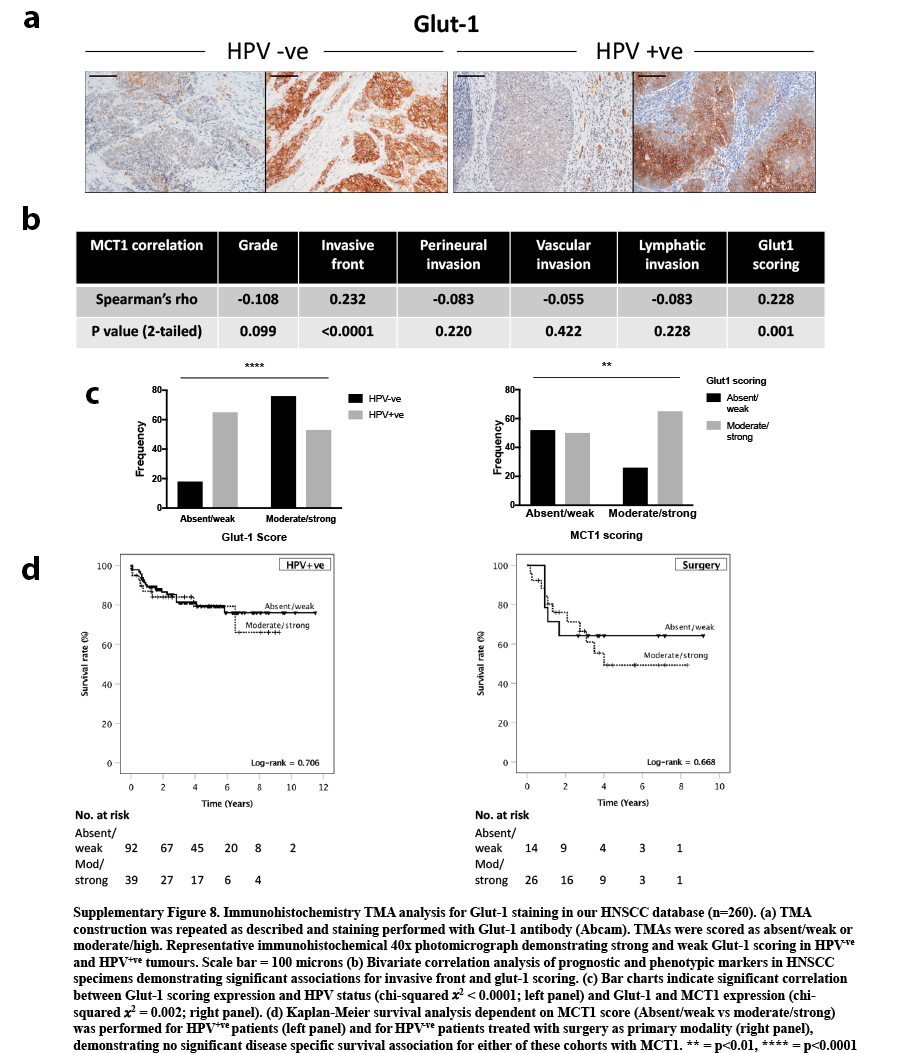

Supplement: Supplementary file 9 — Supplemental Figure 8 [file 41416_2018_364_MOESM9_ESM.tif]

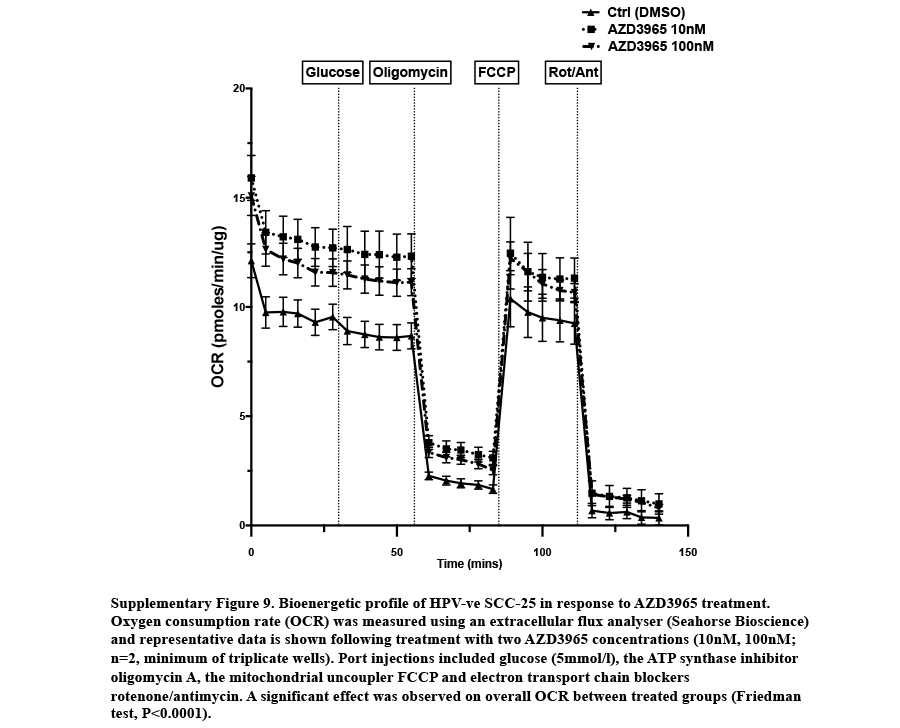

Supplement: Supplementary file 10 — Supplemental Figure 9 [file 41416_2018_364_MOESM10_ESM.tif]
