# Supplementary material for: HPV, tumour metabolism and novel target identification in head and neck squamous cell carcinoma
Source: Br J Cancer. 2019 Jan 17;120(3):356–67. doi: 10.1038/s41416-018-0364-7 (PMC6353968; doi:10.1038/s41416-018-0364-7)
Supplement: Supplementary file 1 — Signed letter approving additional author [file 41416_2018_364_MOESM1_ESM.docx]

06/11/2018

Dear Professor Goding,

Re: **HPV, tumour metabolism and novel target identification in head and neck squamous cell carcinoma**

*Fleming JC, Woo J et al*

We the authors confirm the addition of Dr. Massimiliano Mellone as a co-author to the above paper due to his contribution to the revised paper editing, further results analysis and further experiments performed as requested by the reviewers.

Jason Fleming	…………………………………….

Jeongmin Woo	………… ………………………….

Karwan Moutasim	…………………………………….

Massimiliano Mellone…………………………………….

Steve Frampton	…………………………………….

Abbie Mead	…………………………………….

Waseem Ahmed	…………………………………….

Oliver Wood	…………………………………….

Hollie Robinson	………………………………….

Matthew Ward	…………………………………….

Christopher H. Woelk…………………………………….

Christian H. Ottensmeier…………………………………….

Emma King	…………………………………….

Dae Kim	…………………………………….

Jeremy P Blaydes	…………………………………….

Gareth J. Thomas	…………………………………….
